# Multimodal neuroimaging correlates of physical-cognitive covariation in Chilean adolescents. The Cogni-Action Project

**DOI:** 10.1016/j.dcn.2024.101345

**Published:** 2024-01-17

**Authors:** Carlos Cristi-Montero, Heidi Johansen-Berg, Piergiorgio Salvan

**Affiliations:** a Wellcome Centre for Integrative Neuroimaging, FMRIB Centre, Nuffield Department of Clinical Neurosciences, University of Oxford, John Radcliffe Hospital, Oxford OX3 9DU, United Kingdom; b IRyS Group, Physical Education School, Pontificia Universidad Católica de Valparaíso, Valparaíso 2340025, Chile

**Keywords:** Exercise, Brain, Lifestyle, Multimodal MRI, Canonical correlation analysis, Socioeconomic factors

## Abstract

Health-related behaviours have been related to brain structural features. In developing settings, such as Latin America, high social inequality has been inversely associated with several health-related behaviours affecting brain development. Understanding the relationship between health behaviours and brain structure in such settings is particularly important during adolescence when critical habits are acquired and ingrained. In this cross-sectional study, we carry out a multimodal analysis identifying a brain region associated with health-related behaviours (i.e., adiposity, fitness, sleep problems and others) and cognitive/academic performance, independent of socioeconomic status in a large sample of Chilean adolescents. Our findings suggest that the relationship between health behaviours and cognitive/academic performance involves a particular brain phenotype that could play a mediator role. These findings fill a significant gap in the literature, which has largely focused on developed countries and raise the possibility of promoting healthy behaviours in adolescence as a means to influence brain structure and thereby cognitive/academic achievement, independently of socioeconomic factors. By highlighting the potential impact on brain structure and cognitive/academic achievement, policymakers could design interventions that are more effective in reducing health disparities in developing countries.

## 1 Introduction

Behaviours emerge in response to particular situations, experiences, or repetitive stimuli which modulate our brain through an adaptive process (Zatorre et al., 2012). Although this process occurs throughout the lifespan (Stillman et al., 2020), adolescence is key, as it is a critical period both for acquiring and ingraining habits and for extensive neural reorganisation (Stillman et al., 2020, Dow-Edwards et al., 2019, Dick and Jane Ferguson, 2015).

Adolescents’ physical activity, physical fitness, diet, sleep and other modifiable lifestyle factors have been extensively studied in relation to health outcomes (Jirout et al., 2019, Iso-Markku et al., 2020). However, beyond physical health outcomes, cognitive performance and academic achievement have been recently declared critical outcomes for children and adolescents by the World Health Organisation (Chaput et al., 2020). In this context, health-related behaviors during childhood are crucial due to their profound impact on both the macrostructure (e.g., shape, size, perfusion) and microstructure (e.g., hippocampal neurogenesis, release of neurotrophic factors) of the brain, as well as on cognitive functions, learning abilities, and academic achievements (Jirout et al., 2019, Iso-Markku et al., 2020, Adelantado-Renau et al., 2022, Caamaño-Navarrete et al., 2021, Aghjayan et al., 2021, Walsh et al., 2018, Batouli and Saba, 2017). However, several studies and reports show a low proportion of adolescents meeting the current physical activity and sleep recommendations, a high prevalence of obesity, and a poor diet (Dick and Jane Ferguson, 2015, Walsh et al., 2018, Aguilar-Farias et al., 2020, Schneider et al., 2017); thus creating a potential risk for unhealthy brain and cognitive development.

In addition, socioeconomic factors (including parent education, school vulnerability, and others) play a critical role not just in cognition and academic achievement, where greater deprivation is associated with worse outcomes, but also in brain development (Rakesh and Whittle, 2021, Donofry et al., 2021). In this sense, developing countries and nations with high heterogeneity in income distribution present a significant challenge to reducing socioeconomic inequalities in health (Bor et al., 2017, Eozenou et al., 2021). Nonetheless, health-related behaviours such as physical fitness and physical activity could be leveraged as behavioural drivers to mitigate long-term consequences of early life adversity (i.e., poverty) on brain health (Donofry et al., 2021, Cristi-Montero et al., 2021).

Although social determinants could modulate health-related behaviours and, in turn, brain, cognitive, and academic achievements in adolescents (Cristi-Montero et al., 2021, Lemes et al., 2021, Viner et al., 2012), most literature in this area comes from developed countries, being practically non-existent in children and adolescents living in Latin America (Wassenaar et al., 2020). Chile is a particularly interesting case to study due to it progressively transitioning from low-to-middle to being a high-income country whilst retaining high inequality of income distribution (44.4%). Furthermore, its educational system segregates students into public, subsidised or private schools according to household income (Taut et al., 2009). Therefore, understanding whether health-related behaviours (i.e., fitness, physical activity and, more generally, healthy-lifestyles) relate to brain and cognitive health in adolescence, even in the presence of high heterogeneity in socioeconomic factors, is key to identifying modifiable lifestyle factors capable of improving developmental outcomes in the transition to adulthood.

Human lifestyles are complex, with multiple factors interacting and affecting each other simultaneously. Hence studying the association between individual variables risks missing the numerous interactions among them (Gomersall, 2018, Yeater et al., 2015). Previous studies using multivariate statistical approaches have shown a heterogeneous contribution of each lifestyle factor with brain health indicators in adolescents (i.e., cognitive performance and mental health) (Lemes et al., 2021, Wheatley et al., 2020) and diffuse brain-wide correlates (e.g., mainly structural and microstructural MRI metrics) (Salvan et al., 2021).

Building on this work, here we tested the hypothesis that in a large sample of Chilean adolescents, healthy-lifestyle behaviours (i.e., adiposity, fitness, sleep problems and others) were related to individual differences in cognitive skills and academic achievement (i.e., working memory, attention, maths, language, science), independent of socioeconomic factors, schools, and other confounds of no interest. In a sub-sample with neuroimaging, we also hypothesised that such behaviour–cognitive covariation may have macro and microstructural brain correlates and that these brain correlates may mediate the covariation between health-related behaviours and cognitive/academic achievements, independent of socioeconomic background.

## 2 Methods

This cross-sectional study is part of the Cogni-Action Project (Solis-Urra et al., 2019) carried out from March 2017 to October 2019. The project was conducted according to the guidelines of the Declaration of Helsinki and approved by the Bioethics and Biosafety Committee of the Pontificia Universidad Católica de Valparaíso (BIOEPUCV-H103–2016) and was retrospectively registered (8/July/2020) in the Research Registry (ID: researchregistry5791). Written consents were obtained before participation from the school principal, parents, and participants.

### 2.1 Participants

A total of 1296 adolescents (10–14-years-old, 50% girls) from 19 public, subsidised, and private schools of the Valparaiso region (Chile) participated in this study (Table 1). A subsample of 76 participants was recruited voluntarily to be part of the neuroimaging measurements (magnetic resonance imaging (MRI) scan). Exclusion criteria for neuroimaging analysis were: a) no T1 image (n = 13), b) incidental tumour finding (n = 1), c) no diffusion tensor imaging (DTI) or image quality concern (n = 5). Thus, a total of 57 right-handed adolescents (26 girls, 47%) from 10 schools were included in the neuroimaging analysis. The power sample estimation for the two parts of this study can be found in our protocol paper (Solis-Urra et al., 2019).

**Table 1:** Descriptive participants’ characteristics.

	**Total sample (n = 1296)**	**Neuroimaging subsample (n = 57)**
**Variables**	**Mean (SD) / Frequency (%)**	
Age (years)	11.9 (1.2)	11.6 (1.1)
Sex Girls Boys	648 (50%) 648 (50%)	31 (54%) 26 (46%)
Peak Height Velocity (maturation)	-0.41 (1.3)	-0.67 (1.1)
Socioeconomic factors		
Parent education		
None	869 (67.1%)	25 (43.9%)
Only one	242 (18.7%)	16 (28.1%)
Both	185 (14.3%)	16 (28.1%)
School type		
Public	456 (35.2%)	15 (26.3%)
Subsidised	514 (39.7%)	26 (45.6%)
Private	326 (25.2%)	16 (28.1%)
School Vulnerability Index		
Low	326 (25.2%)	16 (28.1%)
Middle	360 (27.8%)	23 (40.4%)
High	610 (47.1%)	18 (31.6%)

Values are displayed as mean or frequency and SD or percentage, respectively, according to data features. Parent education level is categorised according to the number of parents holding a university degree.

### 2.2 Socioeconomic factors

Socioeconomic factors are crucial during brain development and have been associated with healthy behaviours, cognitive performance and academic achievements (Cristi-Montero et al., 2021, Ursache and Noble, 2016, Huppertz et al., 2017, Hernández-Jaña et al., 2021). We considered multiple measures of socioeconomic factors. First, we included a measure of parental education level categorised according to the number of parents holding a university degree as a) none, b) one, c) both (Huppertz et al., 2017). Second, we included two school-based indicators as school characteristics seem to be a better predictor of adolescents’ cognitive and school performance in Latin America than socioeconomic status (Flores-Mendoza et al., 2018). The first was school type (public, subsidised, or private) because of its good relationship with cognition, health behaviours, and academic performance and its accuracy as an indicator of socioeconomic and parental education levels in the Chilean context (Carrasco and Gunter, 2019). The second was the school vulnerability index, a Chilean metric that measures the degree of socioeconomic vulnerability of pupils who attend schools with partial or total state funding (subsidised and public schools, respectively) based on multiple domains such as: Children Health, Children Education, Habitat and Housing, Family environment and social relationships, Civic Participation, Rights, and Civil Liberties, Family Socioeconomic Context (i.e., socioeconomic condition of the household, unemployment), Communal Socioeconomic Context, and Special Protection and Reparation Measures. This index score ranges from 0 to 100, with a score of zero being assigned to private schools; thus, schools are classified as low (<10), middle (≥10 to <60), and high (≥60) (Hernández-Jaña et al., 2021).

### 2.3 Health-related behaviours measurements

All measurements were carried out in schools, during two four-hour visits per school, separated by eight days. In the first visit, cognitive performance and anthropometry tests were taken, whereas physical fitness was evaluated during the second visit. Cognitive performance and physical fitness were evaluated first in the morning, and questionnaires were administered afterwards.

As part of health-related behaviours, we aimed to measure different lifestyle components either via objective measures or by self-reported questionnaires (Table 2). Body composition was measured by a) body mass index (BMI), b) waist-to-height ratio (WHtR), and c) four skinfolds. Details on the procedure for acquiring these measurements have been reported elsewhere (Hernández-Jaña et al., 2021). Physical activity (self-reported) was evaluated through two measures: a) active commuting and b) physical activity level. The first was evaluated by a question from the Youth Activity Profile questionnaire (YAP-SL) (Segura-Díaz et al., 2021), whilst the second measure was acquired via a validated Chilean questionnaire (Godard M et al., 2008). Physical fitness was assessed through the ALPHA fitness test battery, which evaluates three main fitness components a) muscular fitness (the maximum handgrip strength test plus the standing long jump test), b) cardiorespiratory fitness, and c) speed/agility fitness (Ruiz et al., 2011).

**Table 2:** Descriptive Health-related behaviours measurements.

	**Total sample (n = 1296)**	**Neuroimaging subsample (n = 57)**
**Variables**	**Mean (SD) /** **Frequency (%)**	
Body composition		
Body Mass Index	21.5 (3.8)	21.3 (3.5)
Waist-to-Height Ratio	0.46 (0.1)	0.45 (0.1)
Sum of four skinfolds	65.1 (27.4)	61.4 (22.1)
Physical activity		
Physical activity score	4.7 (1.3)	4.8 (1.5)
Active commuting		
No (none)	916 (70.7%)	46 (80.7%)
Only one way	165 (12.7%)	5 (8.8%)
Yes (both)	215 (16.6%)	6 (10.5%)
Physical fitness components		
Muscular fitness		
Handgrip strength test (kg)	23.0 (6.4)	21.8 (5.7)
Standing long jump test (cm)	141.8 (27.3)	144.0 (22.3)
Cardiorespiratory fitness (min)	4.0 (2.2)	4.0 (2.1)
Speed and Agility fitness (s)	13.0 (1.3)	12.8 (1.2)
Diet		
Mediterranean Score	8.6 (2.2)	9.1 (2.0)
Having Breakfast before the cognitive test		
No	367 (28.3%)	16 (28.1%)
Yes	929 (71.7%)	41 (71.9%)
Breakfast quality score	2.5 (0.9)	2.4 (0.8)
Sleep problems		
Sleep Self-Report Score	12.4 (5.5)	10.8 (5.1)

Values are displayed as mean or frequency and SD or percentage, respectively, according to data features.

The 4 × 10-m shuttle run test was used to assess speed-agility fitness. This test evaluates the speed of movement, agility, and coordination by having participants run as quickly as possible between two lines of cones while carrying a cloth between them. Time was multiplied by − 1, so a higher score indicates better performance. Finally, a z-score based on sex and age was created as a normalised speed-agility fitness score.

The strength of the upper and lower limbs was evaluated as an indicator of muscular fitness. On the one hand, the maximum handgrip strength was assessed using a dynamometer (Jamar Plus+ Digital Hand Dynamometer, Sammons Preston) that was previously adjusted to the child's hand size, allowing for measurements of 0–90 kg with a 0.1 kg precision. This test was performed twice (both hands) in a standing position with the elbow fully extended, and the maximum score between the two measurements was used. To create a relative measure of upper limb strength, the score was divided by body weight. On the other hand, lower limb strength was assessed through the standing long jump test. A starting line was fixed on the floor, and children had to stand with their feet parallel behind the line. At the verbal signal, children had to jump as far as possible, starting with and landing on both feet at the same time. This test was performed twice (with at least 1-min of rest between them), and the longest jump was recorded in centimetres. Finally, the muscular fitness score was created based on the sum of the sex- and age-standardised values of the handgrip/weight and standing long jump.

The cardiorespiratory fitness of the children was evaluated using the 20-meter shuttle run test, which was conducted at the end of the evaluation session. The children were grouped into groups of 8 to 10 and positioned at the starting line. A sound signal was used to indicate the running rhythm, which started at a speed of 8.5 km/h and increased by 0.5 km/h every minute. As a result, the children had to run 20 m and wait on the second line until the next sound signal. To ensure a gradual increase and proper adaptation to the test, a physical education teacher ran alongside the children, guiding them for the first two minutes of the test. The test ended voluntarily when the child was fatigued or unable to reach the line twice. The total time (in seconds) and the number of completed stages were recorded, as recommended. A z-score of the total time (s) based on sex and age was created as a normalised cardiorespiratory fitness score.

Each component was then Z-scored and adjusted for age and sex. Diet was evaluated using three indicators (self-reported): a) adherence to the Mediterranean diet (Serra-Majem et al., 2004), b) having breakfast on the day that cognitive tests were carried out (see below), and c) quality of breakfast (what ingredients were or were not present, e.g. dairy products, cereals, bread and fruits, and fruit juice). In the case of the last two measures, they were included based on their relationship to cognitive performance in this sample of adolescents (Peña-Jorquera et al., 2021). Finally, sleep problems were quantified via the Spanish version of the Sleep Self-Report Questionnaire (Orgilés et al., 2013).

### 2.4 Cognitive and academic achievements

We sought to capture differences in pupils’ cognitive skills and academic achievements. (Table 3). The adolescents’ cognitive performance was evaluated through eight neurocognitive tasks from the NeuroCognitive Performance Test (NCPT) from Lumos Labs, Inc (Morrison et al., 2015). This battery included: “Trail Making A and B” assessing attention, cognitive flexibility, and processing speed; the “Forward Memory Span'' and the ”Reverse Memory Span” evaluating short-term visual memory and working memory; the “Go/No-Go” test, assessing inhibitory control and processing speed; the “Balance Scale,” indicating quantitative and analogical reasoning; the “Digit Symbol Coding,” evaluating processing speed; and finally, the “Progressive Matrices,” assessing problem-solving and reasoning/intelligence (Cristi-Montero et al., 2021). Each test was scaled following a normal inverse transformation of the percentile rank (Morrison et al., 2015).

**Table 3:** Descriptive cognitive and academic achievements.

	**Total sample (n = 1296)**	**Neuroimaging subsample (n = 57)**
**Variables**	**Mean (SD)**	
Cognitive performance		
Trail making test A (Reversed)	100.0 (14.7)	103.6 (15.7)
Trail making test B (Reversed)	100.0 (14.7)	105.1 (13.2)
Forward memory span	100.0 (14.4)	101.0 (12.7)
Reverse memory span	100.0 (14.3)	103.2 (13.1)
Go/No-Go (Reversed)	100.0 (14.7)	101.1 (15.4)
Balance Scale	100.1 (14.5)	104.3 (13.5)
Digit Symbol Coding	100.0 (14.7)	100.3 (15.2)
Progressive Matrices	100.1 (14.3)	101.2 (13.3)
Academic achievement		
Language (grades)	5.40 (0.8)	5.59 (0.8)
Mathematics (grades)	5.36 (1.0)	5.59 (0.9)
Science score (grades)	5.48 (0.8)	5.68 (0.8)

Values are displayed as mean and SD according to data features. Reverse-scored measures had reaction time as the dependent variable.

The data on the adolescent’s grade point average scores (i.e., language, maths, and science) at the end of the school year were taken from the school records. In Chile, the grade scoring range is between 1 to 7 points. These three subjects are part of the Programme for International Student Assessment.

### 2.5 MRI acquisition

All images were obtained with a 1.5 Tesla MRI scanner (AVANTO, Siemens Medical Systems, Erlangen, Germany). Structural MRI: T1 weighted (T1w) three- dimensional rapid gradient echo sequence (3D MPRAGE): TR = 2200 ms; TE = 2,6 ms; flip angle = 8°; FOV = 250 mm; voxel size: 1 × 1 x 1 mm. Sequence duration: 4 min 32 s. Diffusion-weighted MRI (DW- MRI): EPI 2d sequence; b values = 0, 1000 s/mm2, with 20 diffusion- weighted directions; TR = 3300 ms; TE 86 ms; voxel size: 1.8 × 1.8 × 5.0 mm; multiband acceleration GRAPPA factor = 2. Sequence duration: 4.02 min. The entire procedure can be reviewed elsewhere (Solis-Urra et al., 2019).

### 2.6 Structural MRI processing

Preprocessing of T1w-MRI data was performed using FSL BET (Smith, 2002) and the FSL Voxel Based Morphometry (VBM (Smith et al., 2004)) pipeline (https://fsl.fmrib.ox.ac.uk/fsl/fslwiki/). Structural images were brain extracted, and tissue segmentation was performed (Smith, 2002). After quality control, images were aligned to the MNI152 standard-space T1 template using an optimised combination of linear and non-linear registration (FLIRT followed by FNIRT) (Jenkinson et al., 2002, Greve and Fischl, 2009). A second quality-controlled step ensured there was good alignment to standard space for all subjects. For each subject, we quantified both a voxel-based morphometry feature map of grey-matter (VBM; quantifying regional grey matter density) and a voxel-wise map of the Jacobian deformation (JD; measuring local volume differences). Together, these metrics quantify macroscopic brain differences in grey-matter volume across participants. Finally, these images were concatenated into two 4D images and fed to group-level statistics.

### 2.7 *Diffusion MRI processing*

DWI-MRI data was corrected for between-volumes head-movement and eddy currents using FSL Eddy (Andersson and Sotiropoulos, 2015). No reverse phase-encoded image was acquired. Diffusion tensor imaging (DTI) fitting was conducted with FSL DTIFIT (Behrens et al., 2007). FA (fractional anisotropy; quantifying the degree of anisotropic diffusion in water molecules), MD (mean diffusivity; quantifying the total amount of diffusion) and MO (mode of anisotropy; quantifies the type of shape of the DTI ellipsoid) images were then fed into the TBSS pipeline (Behrens et al., 2007, Smith et al., 2007) creating a study-specific, mean FA skeleton, following the procedures used in Salvan et al., (Salvan et al., 2021). Together, these metrics quantify brain differences in white-matter microstructure across participants. These images were then fed to group-level statistics.

### 2.8 Statistical analysis

**Fig. 1:**
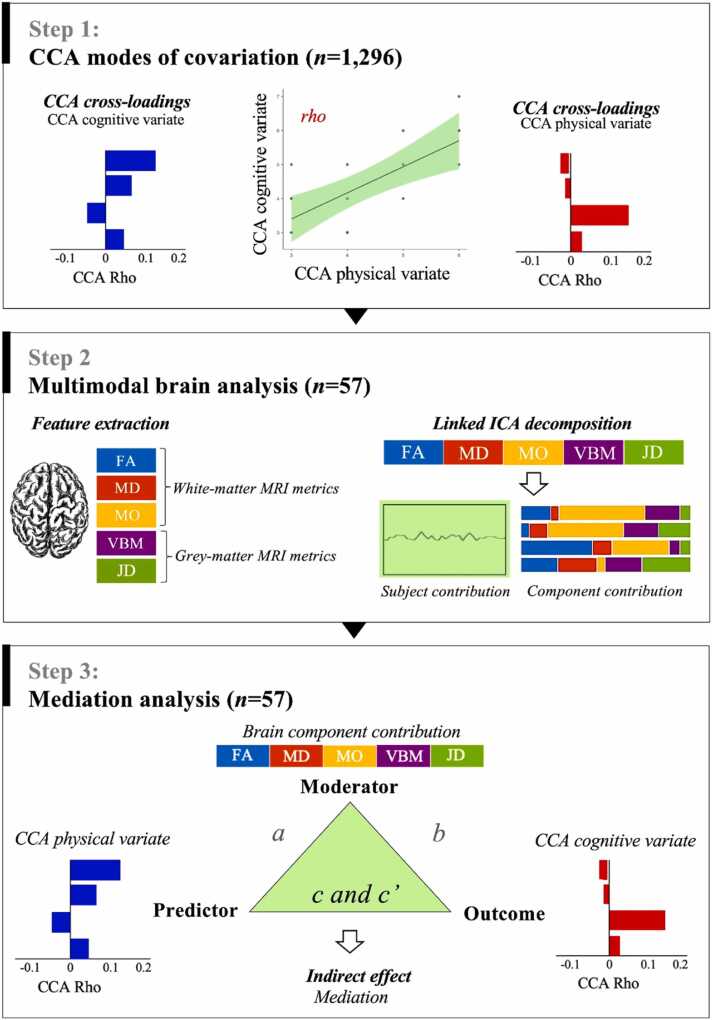
Schematic illustrating the analysis steps. CCA: Canonical correlation analysis; CCA Rho: correlation; FA: Fractional anisotropy; MO: Mode of anisotropy; MD: Mean diffusivity; VBM: Voxel-based morphometry; JD: Jacobian deformation; ICA: independent component analysis; a: equation between predictor and moderator; b: equation between moderator and outcome; c: total effect; c’: direct effect.

A study schematic model is presented in Fig. 1. The first step was to identify modes of covariation between physical and cognitive/academic variables via canonical correlation analysis (CCA). The second step was to characterise independent components of brain structure and microstructure in the sub-sample of pupils with neuroimaging and to test the association with CCA modes. Finally, the third step was to run a mediation analysis to test whether brain components mediate the relationship between physical and cognitive variables.

### 2.9 Imputation

Prior to all statistical testing, any missing data were imputed based on the nonparametric missing value method using random forest through the “missForest” R package (Stekhoven and Bühlmann, 2012). This function successfully imputes large and complex mixed-type datasets (quantitative and/or categorical variables), including complex interactions and non-linear relations by a random forest trained on the observed values predicting the missing values. Prior to imputation, amounts of missing data ranged between 1.2% (i.e., PHV and BMI) to 42.1% (i.e., parental education).

### 2.10 Confounds

Seven covariates were included as confound regressors in our analyses. Age, sex, and maturation are relevant factors associated with behaviour and brain development (Stillman et al., 2020, Lloyd et al., 2014). The differences between chronological and biological age could be reflected in both brain development and cognitive abilities (Brown et al., 2012). Hence, we calculated as a maturity indicator the peak height velocity (PHV), subtracting the PHV age from the chronological age (Moore et al., 2015). In addition, parental education, school type, and school vulnerability index (described above) were included as confounds. Finally, analyses including brain metrics were adjusted for the total brain volume estimated via FreeSurfer (http://surfer.nmr.mgh.harvard.edu/).

### 2.11 Canonical correlation analysis (CCA)

Using CCA we sought to characterise modes of covariation relating two sets of variables: a) health-related behaviour measurements (12 variables) and b) cognitive and academic achievements (11 variables). This approach identifies modes of covariation between the two sets of variables making no prior assumptions about relationships given the canonical cross-loading or strength of correlation (CCA rho) that each variable exerted on its opposite canonical variate. Each mode is characterised by a *pair* of CCA canonical variates or CCA subject-vectors, that are maximally correlated. The total number of modes generated is always equal to the number of variables in the smaller dataset (*here* 11). To perform CCA we used the script permcca (Winkler et al., 2020) (https://github.com/andersonwinkler/PermCCA) whilst adjusting for confounds of no interest (age, sex, PHV, parental education, school type, and school vulnerability index). The significance of CCA modes was calculated via nonparametric inference testing through 1000 permutations among subjects within schools (*k* = 19), respecting dependencies given by the hierarchical structure of the data (Winkler et al., 2015). Family wise error correction (FWE-corr) was applied across all CCA modes in order to correct for multiple comparisons. For those CCA modes deemed significant at FWE-corr p < 0.05, CCA imaging and physical cross-loadings were then extracted.

Cross-loadings were calculated with Pearson’s correlation as in Salvan et al (Salvan et al., 2021). Although small canonical cross-loadings can influence the statistical model's performance alongside larger ones, to aid the interpretation of CCA mode, we focus on CCA cross-loadings with an r value ≥ 0.10 (indicating a small effect size).

### 2.12 Brain imaging networks via multimodal data fusion

We then aimed to characterise parsimonious multimodal patterns of brain structure and microstructure using the structural and microstructural neuroimaging metrics described above. To do this, we performed a multivariate joint-decomposition called FLICA (FMRIB's Linked Independent Component Analysis, https://fsl.fmrib.ox.ac.uk/fsl/fslwiki/FLICA) (Smith et al., 2004, Groves et al., 2011). FLICA is a Bayesian Independent Component Analysis (ICA) approach for multimodal data fusion. Its main goal is to model the imaging data as a set of interpretable features, most of them characterising biophysically plausible modes of variability across all subjects’ images. Unlike in principal component analysis, the mixing matrix vectors of an ICA are not forced to be orthogonal to each other and thus can explain the common variance of variables external to the ICA (Douaud et al., 2014). FLICA was implemented as described in detail in earlier papers (Douaud et al., 2014, Groves et al., 2012). Here, we ran FLICA on five different neuroimaging metrics (FA, MD, MO, VBM, and JD) with 15 components. Although previous large-scale studies have performed FLICA with a higher dimensionality (e.g., 484 subjects and 70 ICs) (Douaud et al., 2014), here, we chose a smaller number of ICs because of the relatively small sample size of 57 pupils. Hence, we expect to identify coarse structural brain networks with a lower granularity compared with previous studies. To test the robustness of our findings to varying the number of FLICA ICs, we also ran FLICA with 14 and 16 components and repeated all statistical tests.

### 2.13 Testing the association between CCA modes and multimodal neuroimaging networks

We next sought to test the association between inter-subject differences in the identified CCA modes (linking health-behaviour with cognitive and academic variables) and differences in multimodal neuroimaging networks in the subset of pupils with neuroimaging data. We tested the regression between the pairs of CCA canonical variates (for each mode: one behavioural and one cognitive) against inter-individual differences in FLICA ICs subject-scores. This was done via a built-in FLICA algorithm (Smith et al., 2004, Groves et al., 2011) that performs Bonferroni correction across ICs tested and non-imaging measures tested. Thus, reported results are fully corrected for multiple comparisons. However, as this test does not take into account the hierarchical structure represented by schools, to corroborate the findings, we performed mixed-linear models explicitly modelling the effect of schools (as a random effect).

Furthermore, although the CCA modes were covaried for several confounds of no interest, there is no guarantee that CCA variance in the subset of pupils with neuroimaging data is independent of such effect. Hence, we further tested whether such associations were independent of the effect of confounds of no interest (age, PHV, sex, school vulnerability index, parent education, school type, and intracranial volume).

### 2.14 *Causal mediation analysis*

A causal mediation analysis was used to test the mediator role of a brain pattern from the multimodal brain analysis (FLICAs) in the relationship between CCA canonical variate predictor (left CCA variate depicting variance in health-related behaviours) and outcome (right CCA variate depicting variance in cognitive and academic achievements). The analysis was conducted using the R ‘mediation’ function (https://cran.r-project.org). The mediation was based on the Baron-Kenny procedure, and standard errors, confidence intervals, and significance levels were calculated based on a quasi-Bayesian Monte Carlo approximation. Four parameters were estimated a) the direct effect (path c, non-mediated effect of health-related behaviours on cognitive/academic achievements), b) the indirect effect (path ab, mediated effect of health-related behaviours on cognitive/academic achievements), c) total effect (path c’ = ab + c), and d) the proportion mediated (proportion of the total effect mediated via the mediator) (VanderWeele and VanderWeele, 2015).

Because inter-subjects’ variance in CCA mode 2 (*fitness–cognition mode*) was significantly correlated with school vulnerability index and school type (respectively, Pearson’s rho = 0.35, p < 0.01; rho = 0.33, p < 0.05; no associations were found for other confounds, see Fig. S5), causal mediation analysis was performed whilst adjusting for these confounds of no interest.

## 3 Results

### 3.1 Covariation modes link health-related behaviours to cognitive skills and academic achievements, independent of socioeconomic factors

**Fig. 2:**
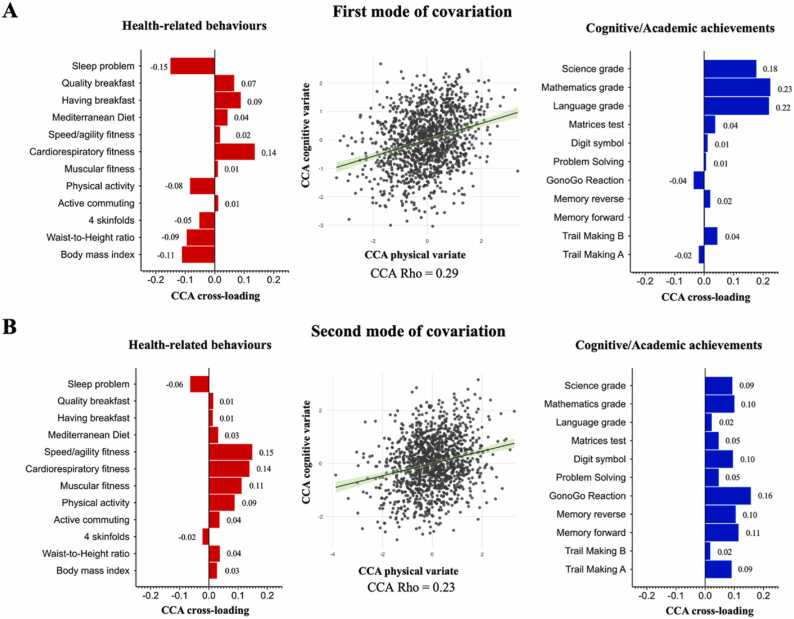
Modes of covariation link sets of health-related behaviours with sets of cognitive/academic variables in adolescents. Showing modes of covariation identified via CCA, adjusted for age, sex, PHV, parental education, school type, school vulnerability index, and school effects. **A)** The first mode shows that pupils with reduced sleep problems, increased cardiorespiratory fitness, and lower body mass index were those who showed greater academic achievement. **B)** Second-mode “physical fitness phenotype”: Pupils with increased physical fitness (all three components) showed greater cognitive skills and match achievements. Of interest, the two modes of covariation are by construction orthogonal, hence independent from each other. Red: CCA cross-loadings for health-related variables. Blue: CCA cross-loadings for cognitive and academic variables. (*n* = 1296). CCA Rho: correlation.

The primary aim of this study was to characterise modes of covariation linking sets of health-related behaviours and cognitive and academic achievements in a large sample of Chilean adolescents, independent of several confounds of no interest. Using CCA, we found two significant modes of covariation. The first mode links all three academic variables positively with cardiorespiratory fitness and negatively with sleep problems and BMI (CCA rho = 0.29, FWE-corr p-value <0.001; Fig. 2**A**). The second mode of covariation links with one academic and four cognitive variables positively with all three physical fitness components (*fitness–cognition mode*; CCA rho = 0.23, FWE-corr p-value <0.001; Fig. 2**B**). Importantly, these modes of covariation are independent of age, PHV, parental education, school administration, and school vulnerability index, and they are present after accounting for the cluster effect of school.

### 3.2 Multimodal brain correlates of fitness–cognitive covariation

**Fig. 3:**
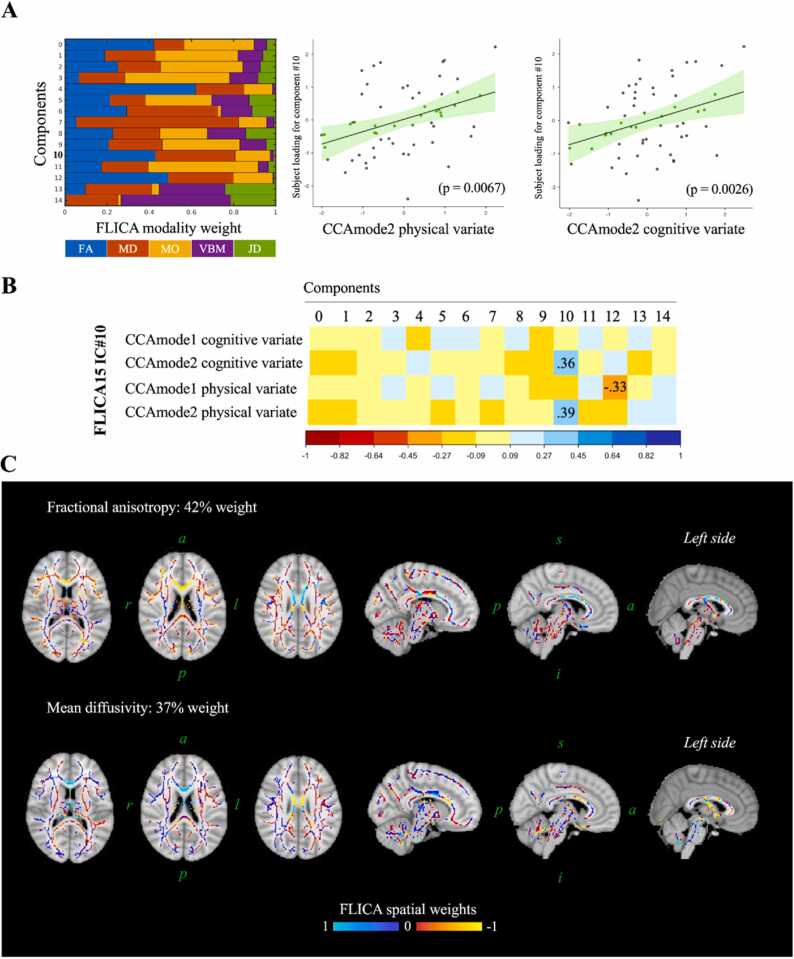
Multimodal neuroimaging correlates of identified covariation modes. **A**) Relative weight of brain features in each component. Left: showing the contribution of each neuroimaging modality to the multimodal ICs (also referred as FLICA modality weights). Centre and right: for sub-set of subjects with MRI data, showing scatter plots of the associations between neuroimaging ICs (FLICA15 IC#10) and CCA physical and cognitive variates for the second mode of covariation. **B**) Showing correlation coefficients between the two identified CCA modes of covariation and neuroimaging ICs as identified by FLICA. Each covariation mode is composed of a pair of CCA variates (see panel **A**). Reporting in numbers only significant associations surviving multiple comparison correction via Bonferroni–thresholdingº Only FLICA15 IC#10 was significantly related to both CCA variates for a given mode. CCACognitive_: CCA cognitive variate. CCAPhysical_: CCA physical variate. **C**) Showing FLICA15 IC#10 brain-wide weights for the two neuroimaging metrics with the highest weights (FA and MD; see panel **A**). *a*: anterior, *p*: posterior, *s*: superior, *i*: inferior, *l*: left, *r*: right. a.u.: arbitrary units.

The secondary aim was to test whether the identified modes of covariation, linking different sets of health-related behaviours with cognitive/academic achievements, are associated with inter-subject variation in multimodal MRI patterns of brain structure and microstructure. Using FLICA, we decomposed inter-subject variation in 5 MRI brain-wide metrics (FA, MD, MO, VBM, and JD) into 15 independent components (FLICA15) or brain networks. Each component is characterised by brain maps of structure and microstructure, depicting the involvement of each MRI metric, and a one-dimensional vector quantifying the subject-wise contribution or, in other words, the subject-scoring along that component. We then used FLICA subject-scorings to test the association between brain structure and microstructure and the identified CCA modes of covariation. After correction for multiple comparisons via a stringent Bonferroni threshold, we found that component IC#10 was significantly associated with both the fitness and cognitive CCA subject-vectors from the second mode of covariation (respectively, p = 0.0067 and p = 0.0026; Bonferroni correction across CCA subject-vectors and FLICA independent components; Figs. 3**A and** 3**B**). These findings show significant brain–behaviour and brain–cognition associations with a brain pattern primarily characterised by white matter microstructure (DTI FA and MD modality weights accounting for ∼80% of FLICA IC#10 variance; see Fig. 3**A**). Specifically, these results show that brain microstructure (higher FA and lower MD in the genu of the corpus callosum) was significantly associated with (i) greater physical fitness (CCA physical variate), as well as with (ii) better cognition and better academic achievement (CCA cognitive variate). This association was robust even after adjusting for confounds of no interest (age, PHV, sex, school vulnerability index, parent education, school type, and intracranial volume) and by using mixed-models accounting for the cluster effect of schools (see Supplementary Table S1). All residuals were normally distributed according to Q-Q plots and the Shapiro-Wilk test (result not shown).

The brain association with the *fitness-cognition mode* was robust even to changing the number of FLICA components. Indeed, both a 14 and 16 factorisation of brain imaging data (respectively, FLICA14 and FLICA16), yielded significant associations with the *fitness-cognition mode* (see Fig. S1; in FLICA16 the association with the CCA cognitive subject-vectors, although significant per se, did not survive correction for multiple comparisons). The inter-subject’s variance of ICs was highly similar to the one characterised by FLICA15 IC#10 (respectively, Pearson’s correlation with FLICA14 IC#8 = 0.89, p-value <0.001; correlation with FLICA16 IC#9 = 0.85, p-value <0.001). Furthermore, the relative contribution of different MRI metrics was also very similar to FLICA15 IC#10 (Fig. 3**A**) (FLICA15 IC#10: 42% FA and 37% MD; FLICA14 IC#8: 45% FA and 45% MD; and FLICA16 IC#9: 47% FA and 38%, Figs. S2 and S3). Crucially, the spatial maps depicting the independent component coefficients matched the overall modality-specific spatial patterns of FLICA15 IC#10, representing high coefficients within Corpus callosum FA and MD (Fig. 3**C and S4**).

One possibility is that the inter-subject variance in brain microstructure pattern of interest is mainly driven by confounds of no interest (such as sex, age, etc.) or by the cluster effect intrinsic in the sampling (the fact that pupils were sampled across different schools, each potentially representing a cluster with different mean). In order to test whether these factors of no interest drive the variance of the component of interest, we performed voxel-wise non-parametric permutation testing, testing the association between FLICA15 IC#10 and inter-subjects’ differences in DTI FA whilst controlling for school vulnerability index and school type and whilst constraining permutations allowing shuffling of samples only within the same school. This analysis showed that corpus callosum’s FA was statistically significantly driving the decomposition of FLICA15 IC#10, even after adjusting for socioeconomic indicators (supplementary material, Fig. S4).

### 3.3 A trend towards mediation in the fitness–cognitive covariation

Finally, we hypothesised that the identified brain microstructural network (FLICA15 IC#10) could mediate the relationship between fitness and cognitive CCA subject-vectors from the second mode of significant covariation, independent of socioeconomic factors (school vulnerability and school type). As the significance of the association between fitness–brain microstructure (*path a*) and brain microstructure–cognition (*path b*) was previously established (Fig. 3**A**), we next performed a causal mediation analysis. We found a trend towards significance for the Indirect effect (*path ab*: p-value = 0.088, 95% CI −0.007 to 0.260; Table 4) with the Total effect losing statistical significance without the presence of the mediator (Total effect c: p-value 0.008; Direct effect c’: p-value = 0.092).

**Table 4:** Causal mediation analysis with FLICA15 IC#10 as mediator between CCA covariates.

	Estimate	95% CI Lower to Upper	p
Indirect effect	0.106	-0.007 to 0.260	0.088
Direct Effect	0.280	-0.038 to 0.580	0.092
Total effect	0.385	0.080 to 0.680	**0.008**
Prop. Mediated	0.26	-0.050 to 1.250	0.096

Sample size: 57, Quasi-Bayesian Confidence Intervals, Simulations: 1000. Significant values are highlighted in bold.

We further tested the robustness of this trend towards a mediation by replicating the causal mediation analysis for FLICA14 IC#8 and FLICA16 IC#9. FLICA14 showed a significant Indirect effect (*path ab*: p-value = 0.010) with the Total effect losing statistical significance without the presence of the mediator (Total effect c: p-value 0.010; Direct effect c’: p-value = 0.090). Whilst FLICA16 showed a trend for the indirect path (*path ab*: p-value = 0.076), the Total and the Direct effects were both statistically significant (p-value = 0.008 and 0.030, respectively) (more details see Tables S2). These findings suggest that the favourable relationship between fitness CCA and cognitive CCA appears to be mediated by the brain's microstructural network, as identified in our FLICA analysis. Specifically, FLICAs 14 and 16 revealed a trend, while FLICA 15 demonstrated a significant indirect effect, indicating a mediation process. Furthermore, the direct and total effects tended to remain significant even after accounting for the mediation influence. This suggests that mediation is only partial and not entirely conclusive, implying the involvement of other brain components in this mediating effect.

## 4 Discussion

### 4.1 Modes of covariation link pupils’ health-related behaviours with cognitive skills and academic achievements

The first aim of this study was to characterise modes of covariation relating sets of health-related behaviours with sets of cognitive and academic achievement variables in a large sample of Chilean adolescents. We found two significant modes of covariation, above and beyond the effect of socioeconomic factors and of other confounds of no interest. The first mode related greater academic achievement with lower sleep problems, higher cardiorespiratory fitness, and lower BMI. The second mode related greater cognitive skills (inhibitory control, processing speed, working memory) and greater maths and science abilities with greater fitness across all physical fitness components tested (cardiorespiratory, muscular, and speed/agility fitness) and with greater physical activity, a phenotype to which we refer to as *physical fitness*. Importantly, these two modes are independent from one another and differ in their overall phenotypes.

Our findings are in line with the literature showing an important contribution of multiple healthy-lifestyle behaviours – such as physical activity, physical fitness, sleep hygiene, diet, body composition – in supporting adolescents’ cognitive performance and academic achievements (Walsh et al., 2018, Hernández-Jaña et al., 2021, Solis-Urra et al., 2021, Tapia-Serrano et al., 2022, Liu et al., 2022). It is indeed well-known that lifestyle behaviours are interdependent, favouring a synergistic beneficial effect on the body (Tremblay et al., 2016). However, here we further unveil that this lifestyle–cognition covariation is characterised by two distinct and independent latent sources, each characterised by distinct physical/behavioural and cognitive/academic phenotypes. This result suggests two distinct pathways that could be leveraged via modifiable behaviours in order to improve cognition and academic achievements during adolescence.

Previous studies examining adherence to 24-h movement guidelines (Walsh et al., 2018, Tapia-Serrano et al., 2022, Liu et al., 2022) (regarding physical activity, sleep, and sedentary behaviour) have found some association between adherence to these guidelines and both cognitive outcomes (4524 US children aged 8–11 years) (Walsh et al., 2018) and academic achievement (1290 Spanish adolescents aged 11–16 years) (Tapia-Serrano et al., 2022, Liu et al., 2022). However, there is heterogeneity in these associations depending on the population studied and which combination of guidelines was considered (Tapia-Serrano et al., 2022, Liu et al., 2022). Therefore, multivariate analyses, integrating more variables reflecting the complexity of human behaviour, might be better suited to identify more robust and plausible modes of variability relating lifestyles and cognition, and hence could provide novel evidence for movement guidelines.

For example, we previously used structural modelling of data from the Chilean Cogni-Action Project, to test the multivariate association among age, health-related quality of life, school vulnerability index, body mass index, physical activity, and sleep problems with physical fitness and cognitive performance in adolescents (Lemes et al., 2021). We found that physical fitness mediated the relationship between school vulnerability index, body mass index, and physical activity and cognitive performance. Using data from the Fit to Study Project in the UK, we previously used CCA to uncover latent factors relating sets of physically active lifestyle measures with mental health and cognition (Wheatley et al., 2020). The results of the current study further emphasise how modifiable behavioural factors, such as health-related lifestyle, may support cognitive development and academic achievements in adolescence, even in the presence of high heterogeneity in the socioeconomic environment.

### 4.2 Multimodal brain correlates of covariation modes

The second aim of this study was to identify the brain correlates of the identified modes of covariation described above in a subgroup of pupils that underwent neuroimaging. We found that pupils’ differences along the *fitness-cognition mode* were significantly associated with a brain microstructure phenotype. Pupils with both greater cardiovascular fitness and greater cognitive skills were those who also exhibited greater FA and lower MD in the corpus callosum, a pattern of neuroimaging metrics often related to better brain health and cognitive development (Douaud et al., 2014). Although this brain network expressed little contribution from brain grey-matter structural metrics, the identified relationship was robust to several confounds of no interest, to the cluster effect due to schools, and to varying the dimensionality of the neuroimaging decomposition method.

There is limited prior literature investigating the multimodal brain correlates of lifestyle behaviours in children and adolescents. Salvan et al. (2021) showed that in a sample of 50 12-year old UK adolescents, a physically active lifestyle (being fitter and less sedentary) was associated with systems-level brain variation across multiple MRI metrics (greater grey-matter perfusion, volume, cortical surface area, greater white-matter extra-neurite density, and resting-state networks activity) (Salvan et al., 2021). Although this relationship was robust to a number of confounds of no interest and suggested the presence of diverse biological processes known to be related to change in fitness in rodent experiments, the Authors did not find a significant association with pupils’ cognitive performance. Our findings complement this previous research by showing that a brain-wide pattern of white-matter microstructure relates to interindividual differences in a number of variables related to a healthy-lifestyle as well as to greater cognitive skills.

Traditionally, hippocampal volume and hippocampal connectivity have been associated with higher cardiorespiratory fitness levels in children and adolescents (Chaddock et al., 2010, Herting and Nagel, 2013, Esteban-Cornejo et al., 2021). However, several studies have also found beneficial effects of physical training programmes on brain white-matter. For instance, an intervention study reported that children participating in exercise (8-month, 40 min of aerobic activities per day) showed improved white-matter microstructure compared to controls (Schaeffer et al., 2014). An after-school program (2 h per day for 150 days of moderate-to-vigorous physical activity) found that children who participated in the physical activity program increased white-matter FA and decreased MD in the genu of the corpus callosum (Chaddock-Heyman et al., 2018). While another study (8-month, 40 min of aerobic exercise per day) showed that participating in an exercise intervention improves white-matter microstructure in children as compared to a sedentary after-school program (Krafft et al., 2014).

Furthermore, previous cross-sectional studies have also reported that physical activity was associated with greater white-matter microstructure (Chaddock-Heyman et al., 2018), and cardiorespiratory fitness was associated with greater white-matter volume (Esteban-Cornejo et al., 2019), microstructure, and connectivity (Chaddock-Heyman et al., 2014, Herting et al., 2014). Therefore, our findings add to this literature by suggesting that modifiable behaviours such as increasing physical activity and physical fitness could help improve brain development and, in turn, cognitive skills in adolescents.

### 4.3 Mediation role of brain microstructure

We finally tested whether brain differences mediated the effect of fitness on cognition. Our finding hints at a possible mediator role of healthy brain microstructure on the *fitness–cognition mode* of covariation, independent of socioeconomic status. The association between white-matter microstructure and either executive functions and academic achievements, or active lifestyle markers (i.e., physical activity and physical fitness), both during childhood and adolescence, is well established from previous studies (Goddings et al., 2021, Till et al., 2011, Ozernov-Palchik et al., 2019, Romeo et al., 2018, Ruotsalainen et al., 2020). Several studies have shown a significant association of cardiorespiratory fitness (Chaddock-Heyman et al., 2014, Herting et al., 2014) and physical activity (Chaddock-Heyman et al., 2018) with higher FA in sections of the corpus callosum. On the other hand, the corpus callosum is a fundamental white-matter bundle that supports executive functions and academic achievements. Indeed better inhibitory control (Fjell et al., 2012), working memory (Bathelt et al., 2018), task-switching (Vallesi et al., 2016), language (Fryer et al., 2008), and maths performance (Till et al., 2011) have been linked to higher FA in the corpus callosum. However, very little evidence exists on the mediator role of brain microstructure in the link between cardiorespiratory fitness and cognitive performance.

To the best of our knowledge, only Ruotsalainen, et al., (2020) and Maijer et al., (2021) have explored FA as a moderator or mediator between physical fitness and neurocognitive functioning indicators. Ruotsalainen, et al., (2020) found that in 12–16 years old adolescents the white-matter microstructure of the corpus callosum moderates the association between cardiorespiratory fitness and working memory (Ruotsalainen et al., 2020). Maijer et al., (2021) found no significant mediation of FA in the relationship between cardiorespiratory fitness and neurocognitive functioning (Meijer et al., 2021). Our finding of a trend for a mediating role of the brain phenotype in the relationship between fitness and cognition builds on this existing literature. It hints that greater *physical fitness* may support neurocognitive development by improving brain microstructure in a white-matter bundle that is fundamental for the integration of information across brain networks and that is a key neural correlate of multiple cognitive functions. However, future studies should replicate this mediation analysis with properly powered sample sizes.

### 4.4 Strengths and limitations

To our knowledge, this is the first study integrating the covariation between health-related behaviours and cognitive/academic variables, with a multimodal neuroimaging analysis in adolescents living in a Latin-American country. These relationships were significant and robust above and beyond the effect of socioeconomic factors, measured via three different variables related to family, school, and social features. This is an important feature of this study that may be of great interest to developing countries and nations with high inequality in income distribution.

While the overall sample in our study was relatively large, our neuroimaging subsample was considerably smaller. In addition, the final sample size for the multimodal neuroimaging analysis had lower statistical power than originally planned (Solis-Urra et al., 2019). Therefore, future studies should assess whether the trend for a mediation found in this work can be detected with a bigger sample size.

## 5 Conclusion

The findings reported in this study are of high relevance to public health and educational policies due to the worldwide decline in children and adolescents’ cardiorespiratory fitness (Eberhardt et al., 2020). This is particularly true in countries with high inequality in income distribution (Tomkinson et al., 2019) and low educational achievement, a phenomenon that has been exacerbated globally over the past 15 years (UNESCO and Global Education Monitoring Report Team, 2020). Our findings show that healthier white-matter microstructure is a clear and robust neural correlate of the relationship between greater cardiorespiratory fitness and higher cognitive performance, independent of effects related to socioeconomic factors. This suggests that promoting health-related lifestyle behaviours could result in a broad brain pattern of microstructural changes that, in turn, may support healthy neurocognitive development in adolescence. Thus, early education focusing on healthy behaviours would be a valuable and low-cost strategy to bridge the cognitive/academic gap due to social inequalities.

## CRediT authorship contribution statement

**CC-M, HJ-B,** and **PS** conceived and designed the data analysis and manuscript. **CC-M** was responsible for coordinating the study, acquiring the data, and writing the first manuscript version. All authors contributed significantly to editing the manuscript and agreed to the final version.

## Declaration of generative AI and AI-assisted technologies in the writing process

During the preparation of this work the author(s) used ChatGPT 3.5 in order to improve language and readability. After using this tool/service, the author(s) reviewed and edited the content as needed and take(s) full responsibility for the content of the publication.

## Declaration of Competing Interest

The authors declare that they have no known competing financial interests or personal relationships that could have appeared to influence the work reported in this paper.

## Data Availability

The data presented in this study are available on request from the corresponding author. The data are not publicly available as we did not obtain consent for public release of data.
