# Supplementary material for: Multimodal neuroimaging correlates of physical-cognitive covariation in Chilean adolescents. The Cogni-Action Project
Source: Dev Cogn Neurosci. 2024 Jan 17;66:101345. doi: 10.1016/j.dcn.2024.101345 (PMC10832367; doi:10.1016/j.dcn.2024.101345)
Supplement: Supplementary file 1 — Supplementary material [file mmc1.docx]

**Supplementary material**

**Figure S1.** Associations between all four modes of covariations and FLICA14, 15, and 16.


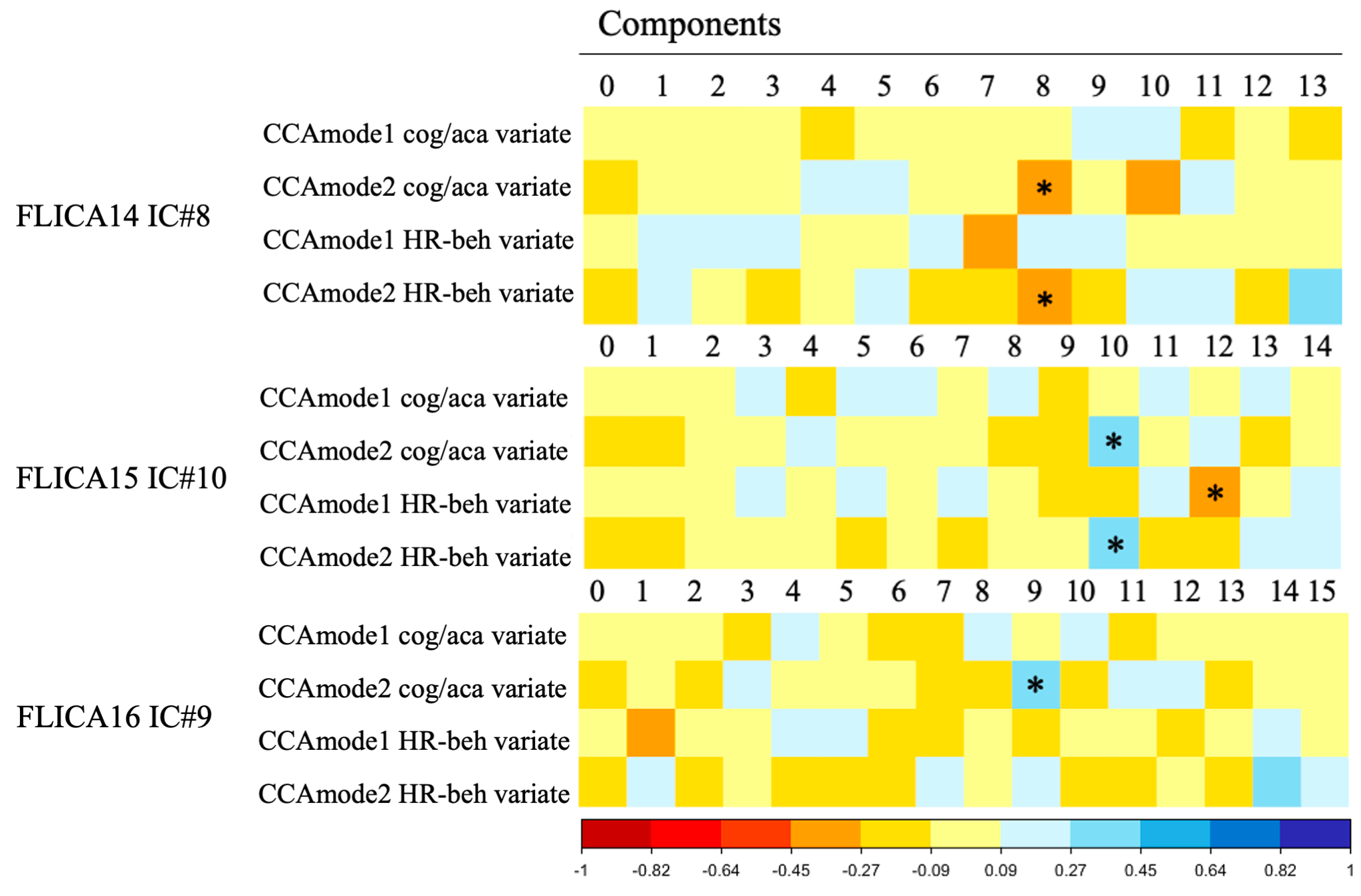


Correlation matrix between all four modes of covariation and FLICA’s components. * significant associations (r) after multiple comparisons. The colour indicates the grade of association.

**Figure S2.** The relative weight of brain features for each component by FLICA and its association with the second mode of covariation.


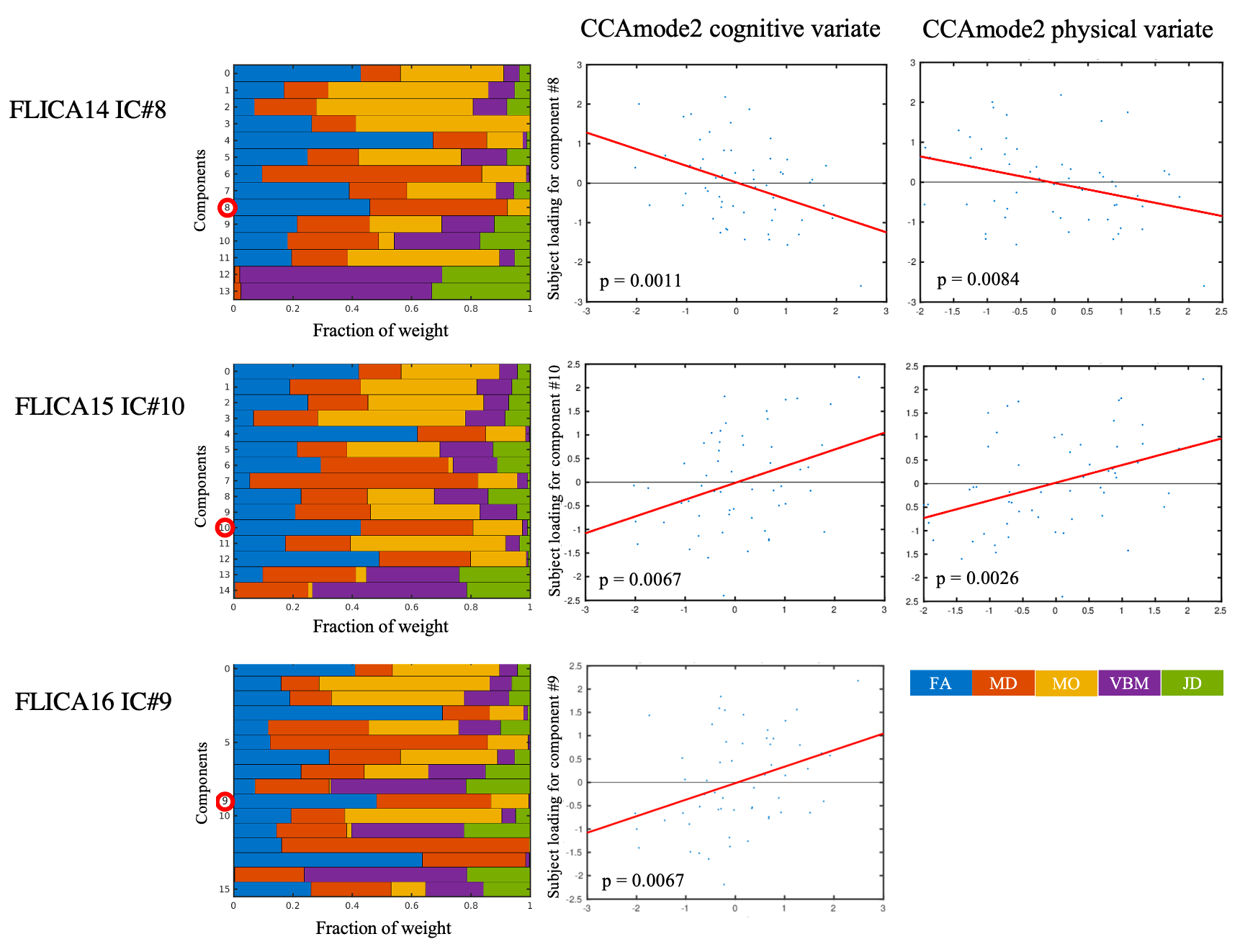


The first column (left) displays the fraction of weight for each component (the red circle represents the component studied). The two columns in the right show the association with the main modes of covariation (cognitive/academic achievements and health-related behaviours, respectively) from the mode of covariation number two. P-value after multiple comparisons.

**Figure S3.** Fractional anisotropy and mean diffusivity relative contributions for main FLICA 14, 15 and 16-components.

**Panel A:** Fractional anisotropy.


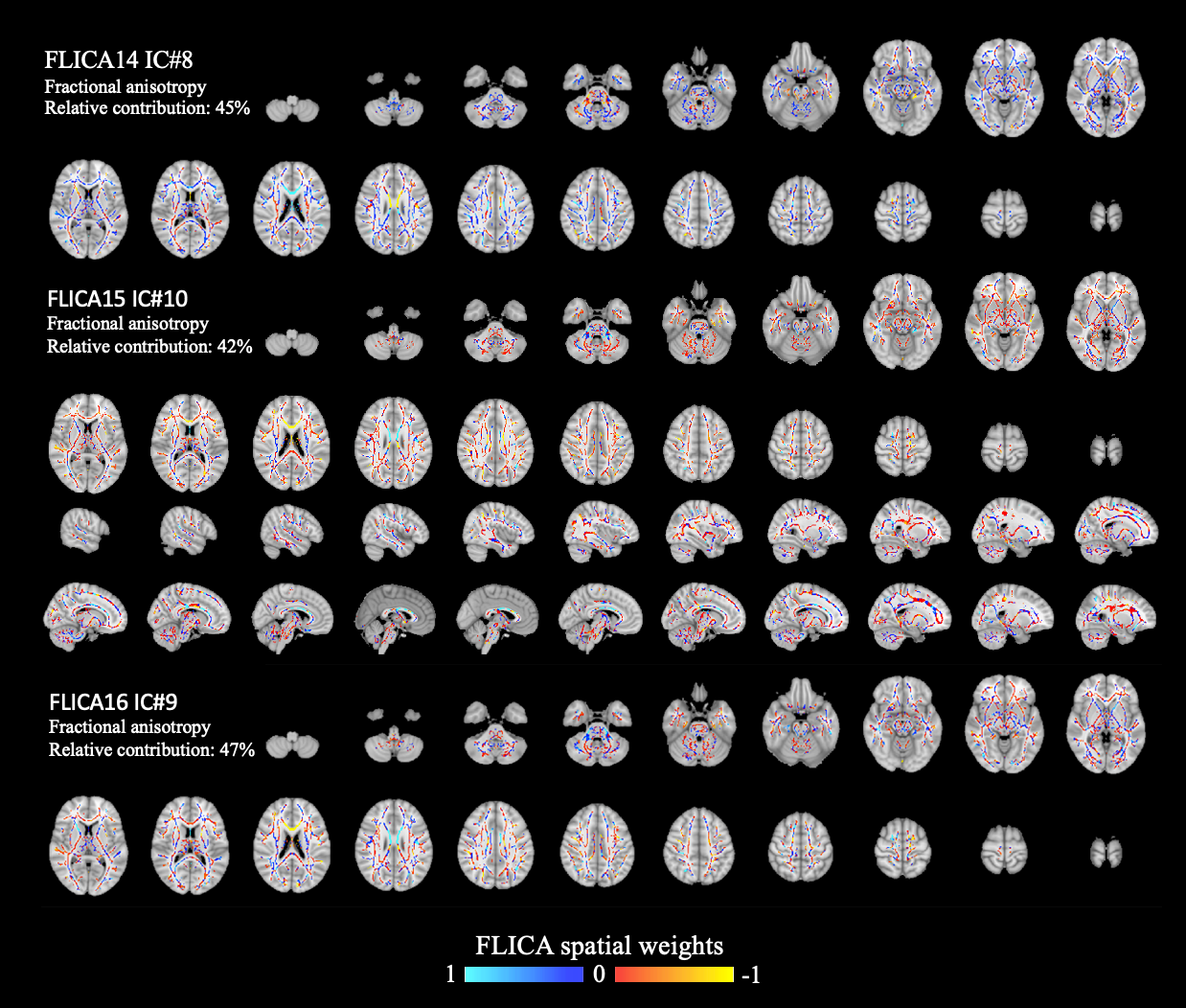


**Panel B:** Mean diffusivity.


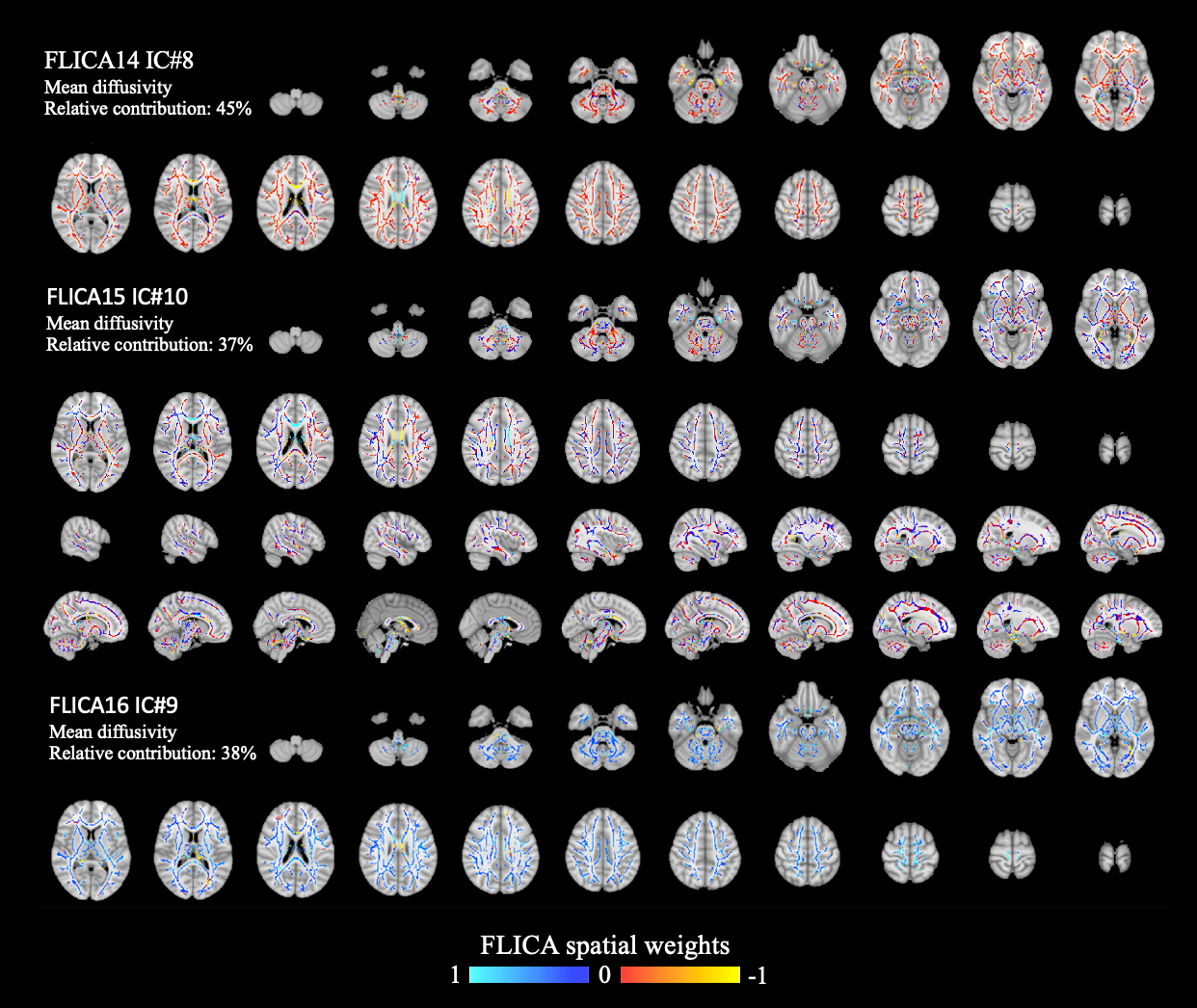


**Figure S4.** Voxel-wise statistics on DTI FA.

**
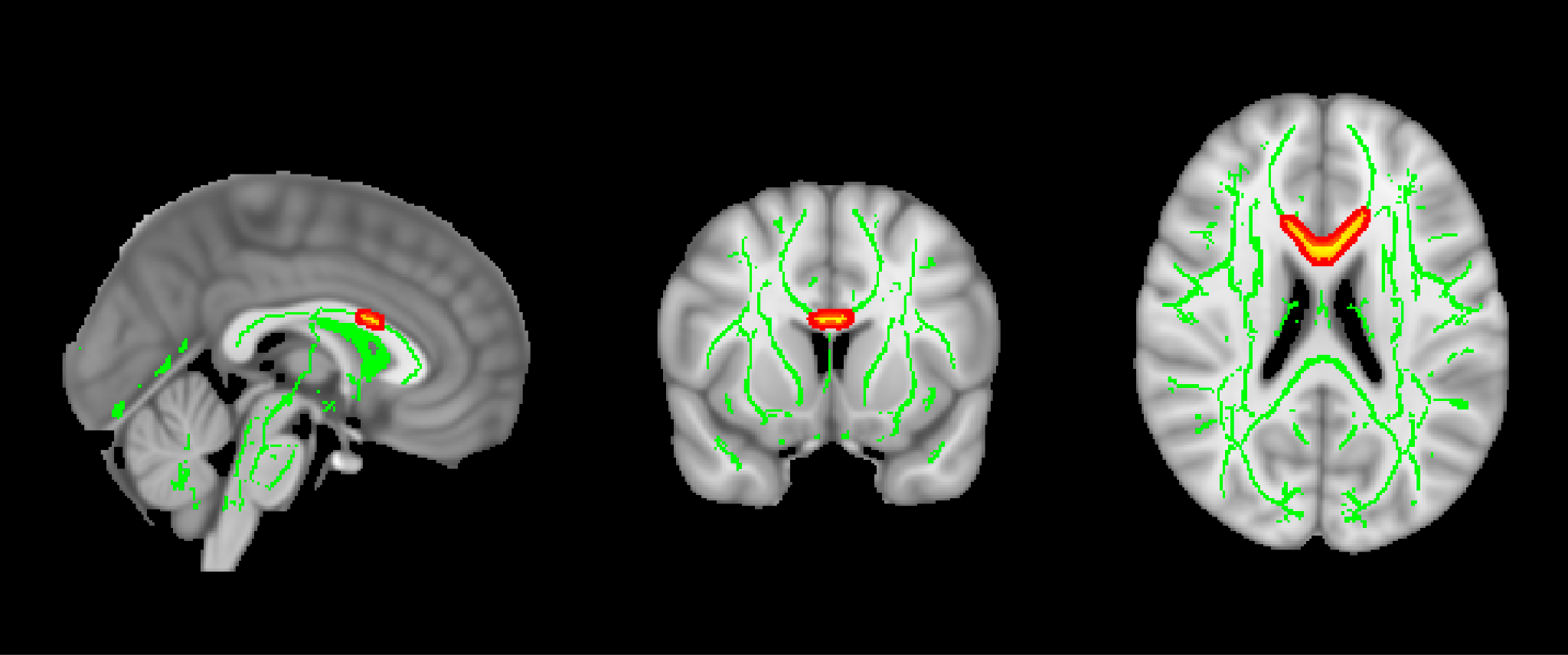
**

Showing voxel-wise statistical map of the non-parametric permutation testing in the linear association between FLICA15 IC#10 and inter-subjects differences in DTI FA, whilst adjusting for school vulnerability index and school type, and whilst constraining permutations allowing shuffling of samples only within the same school. Results are thresholded for visualisation purposes at FWE-corr P < 0.05. Green: Study-specific FA-skeleton created via FSL TBSS. Red-Yellow: significant cluster of association (FWE-corr P < 0.05). The statistical map shows that corpus callosum’s FA inter-subject differences were driving the decomposition of FLICA15 IC#10, even after adjusting for socioeconomic indicators and schools-structure in the sampling.

**Figure S5.** Correlation matrix between the second modes of covariation and all confounds.

**
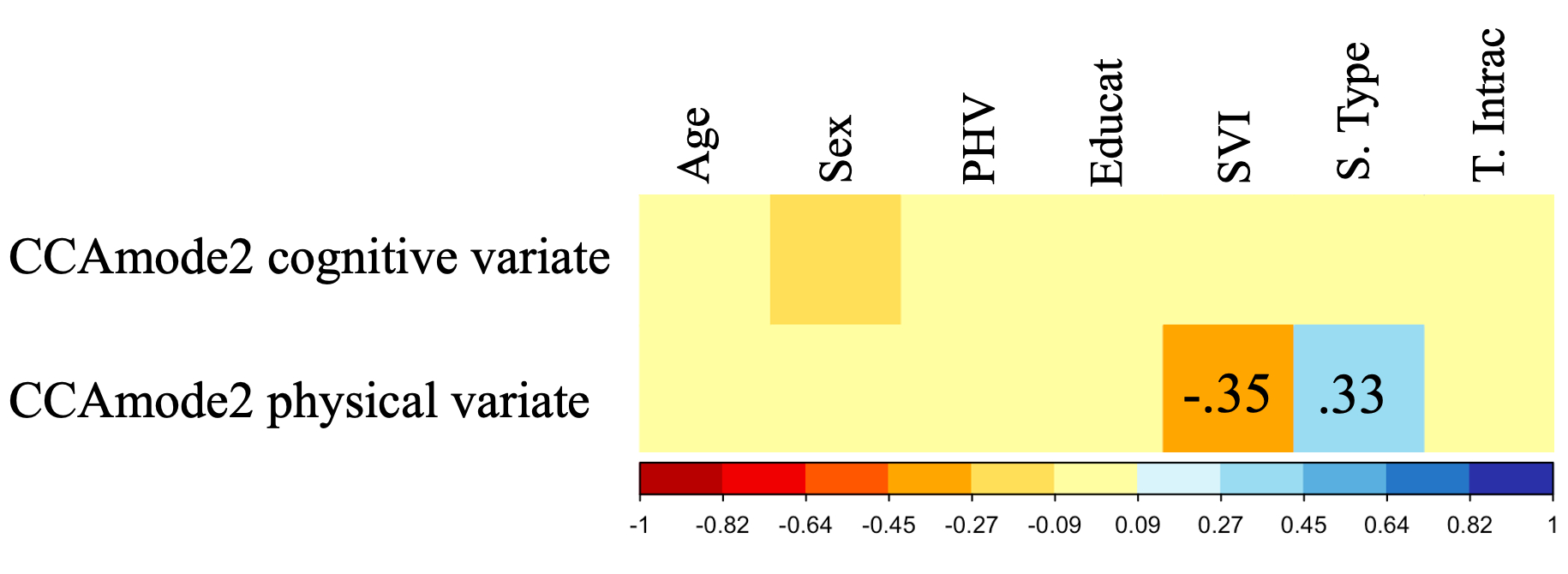
**

The plot displays a correlation matrix between the *fitness-cognition mode* and all confounds used in analyses. PHV: peak height velocity (maturation), Educat: Parental education, SVI: school vulnerability index, S. Type: School type (administration), T. Intrac: total intracranial volume.

**Tables S1.** Mixed-models by modes of covariation (fixed effects parameter estimates).

**Table S1A: FLICA 14 IC#8**

CCAmode2 cognitive variate: Model 1

| **Names** | **Estimate** | **SE** | **Lower** | **Upper** | **df** | **t** | **p** |
| --- | --- | --- | --- | --- | --- | --- | --- |
| (Intercept) | -0.003 | 0.122 | -0.243 | 0.236 | 55.000 | -0.028 | 0.978 |
| CCA_Cogn_mode2 | -0.421 | 0.122 | -0.660 | -0.182 | 55.000 | -3.451 | **0.001** |

CCAmode2 cognitive variate: Model 2

| **Names** | **Estimate** | **SE** | **Lower** | **Upper** | **df** | **t** | **p** |
| --- | --- | --- | --- | --- | --- | --- | --- |
| (Intercept) | -0.003 | 0.130 | -0.258 | 0.251 | 48.000 | -0.026 | 0.979 |
| Intracranial_Vol | 0.030 | 0.138 | -0.239 | 0.300 | 48.000 | 0.221 | 0.826 |
| Parental_educatoin | -0.055 | 0.157 | -0.362 | 0.252 | 48.000 | -0.349 | 0.728 |
| Sex | -0.015 | 0.253 | -0.510 | 0.480 | 48.000 | -0.058 | 0.954 |
| PHV | -0.095 | 0.329 | -0.740 | 0.551 | 48.000 | -0.287 | 0.775 |
| Age | 0.025 | 0.263 | -0.489 | 0.540 | 48.000 | 0.097 | 0.923 |
| SVI | -0.081 | 0.218 | -0.509 | 0.347 | 48.000 | -0.371 | 0.712 |
| School_Type | 0.014 | 0.206 | -0.390 | 0.419 | 48.000 | 0.070 | 0.944 |
| CCA_Cogn_mode2 | -0.437 | 0.133 | -0.697 | -0.176 | 48.000 | -3.287 | **0.002** |

Model 1: no adjusted; Model 2: Adjusted by age, PHV, sex, SVI, parent education, school type, and intracranial volume. Cluster: Schools (*k*=10). 95% Confidence Interval (Lower to Upper). In bold significant values.

**Table S1B: FLICA 14 IC#8**

CCAmode2 physical variate: Model 1

| Names | **Estimate** | **SE** | **Lower** | **Upper** | **df** | **t** | **p** |
| --- | --- | --- | --- | --- | --- | --- | --- |
| (Intercept) | 0.011 | 0.143 | -0.269 | 0.291 | 6.081 | 0.076 | 0.942 |
| CCA_Beh_mode2 | -0.364 | 0.123 | -0.606 | -0.123 | 50.630 | -2.958 | **0.005** |

CCAmode2 physical variate: Model 2

| **Names** | **Estimate** | **SE** | **Lower** | **Upper** | **df** | **t** | **p** |
| --- | --- | --- | --- | --- | --- | --- | --- |
| (Intercept) | 0.016 | 0.156 | -0.291 | 0.322 | 3.110 | 0.102 | 0.925 |
| CCA_Beh_mode2 | -0.481 | 0.144 | -0.764 | -0.198 | 47.964 | -3.330 | **0.002** |
| Intracranial_Vol | 0.008 | 0.138 | -0.262 | 0.278 | 47.177 | 0.060 | 0.952 |
| Parental_educatoin | -0.146 | 0.162 | -0.463 | 0.171 | 47.703 | -0.903 | 0.371 |
| Sex | 0.170 | 0.257 | -0.333 | 0.673 | 45.136 | 0.664 | 0.510 |
| PHV | -0.300 | 0.337 | -0.960 | 0.359 | 47.853 | -0.892 | 0.377 |
| Age | 0.140 | 0.265 | -0.379 | 0.659 | 47.742 | 0.529 | 0.599 |
| SVI | -0.158 | 0.261 | -0.670 | 0.354 | 3.400 | -0.606 | 0.582 |
| School_Type | 0.145 | 0.250 | -0.345 | 0.634 | 3.557 | 0.579 | 0.597 |

Model 1: no adjusted; Model 2: Adjusted by age, PHV, sex, SVI, parent education, school type, and intracranial volume. Cluster: Schools (*k*=10). 95% Confidence Interval (Lower to Upper). In bold significant values.

**Table S1C: FLICA 15 IC#10**

CCAmode2 cognitive variate: Model 1

| **Names** | **Estimate** | **SE** | **Lower** | **Upper** | **df** | **t** | **p** |
| --- | --- | --- | --- | --- | --- | --- | --- |
| (Intercept) | 0.001 | 0.126 | -0.246 | 0.248 | 55.000 | 0.006 | 0.995 |
| CCA_Cogn_mode2 | 0.354 | 0.126 | 0.108 | 0.601 | 55.000 | 2.817 | **0.007** |

CCAmode2 cognitive variate: Model 2

| **Names** | **Estimate** | **SE** | **Lower** | **Upper** | **df** | **t** | **p** |
| --- | --- | --- | --- | --- | --- | --- | --- |
| (Intercept) | 0.001 | 0.132 | -0.258 | 0.260 | 48.000 | 0.006 | 0.995 |
| Intracranial_Vol | -0.108 | 0.140 | -0.383 | 0.166 | 48.000 | -0.775 | 0.442 |
| Parental_educatoin | 0.174 | 0.159 | -0.138 | 0.486 | 48.000 | 1.092 | 0.280 |
| Sex | 0.140 | 0.257 | -0.363 | 0.643 | 48.000 | 0.545 | 0.589 |
| PHV | -0.077 | 0.335 | -0.732 | 0.579 | 48.000 | -0.229 | 0.820 |
| Age | 0.023 | 0.267 | -0.500 | 0.546 | 48.000 | 0.085 | 0.933 |
| SVI | 0.133 | 0.222 | -0.302 | 0.567 | 48.000 | 0.598 | 0.552 |
| School_Type | -0.016 | 0.210 | -0.427 | 0.395 | 48.000 | -0.078 | 0.938 |
| CCA_Cogn_mode2 | 0.381 | 0.135 | 0.116 | 0.646 | 48.000 | 2.820 | **0.007** |

Model 1: no adjusted; Model 2: Adjusted by age, PHV, sex, SVI, parent education, school type, and intracranial volume. Cluster: Schools (*k*=10). In bold significant values.

**Table S1D: FLICA 15 IC#10**

CCAmode2 physical variate: Model 1

| **Names** | **Estimate** | **SE** | **Lower** | **Upper** | **df** | **t** | **p** |
| --- | --- | --- | --- | --- | --- | --- | --- |
| (Intercept) | 0.001 | 0.124 | -0.242 | 0.244 | 55.000 | 0.007 | 0.995 |
| CCA_Beh_mode2 | 0.376 | 0.119 | 0.142 | 0.609 | 55.000 | 3.156 | **0.003** |

CCAmode2 physical variate: Model 2

| **Names** | **Estimate** | **SE** | **Lower** | **Upper** | **df** | **t** | **p** |
| --- | --- | --- | --- | --- | --- | --- | --- |
| (Intercept) | 0.001 | 0.125 | -0.244 | 0.246 | 48.000 | 0.007 | 0.995 |
| CCA_Beh_mode2 | 0.528 | 0.138 | 0.256 | 0.799 | 48.000 | 3.815 | **< .001** |
| Intracranial_Vol | -0.064 | 0.133 | -0.324 | 0.196 | 48.000 | -0.486 | 0.629 |
| Parental_educatoin | 0.284 | 0.155 | -0.021 | 0.588 | 48.000 | 1.827 | 0.074 |
| Sex | -0.087 | 0.242 | -0.562 | 0.387 | 48.000 | -0.360 | 0.720 |
| PHV | 0.190 | 0.321 | -0.439 | 0.819 | 48.000 | 0.592 | 0.557 |
| Age | -0.085 | 0.253 | -0.580 | 0.410 | 48.000 | -0.336 | 0.739 |
| SVI | 0.226 | 0.213 | -0.190 | 0.643 | 48.000 | 1.064 | 0.293 |
| School_Type | -0.195 | 0.204 | -0.596 | 0.205 | 48.000 | -0.957 | 0.344 |

Model 1: no adjusted; Model 2: Adjusted by age, PHV, sex, SVI, parent education, school type, and intracranial volume. Cluster: Schools (k=10). In bold significant values.

**Table S1E: FLICA 16 IC#9**

CCAmode2 cognitive variate: Model 1

| **Names** | **Estimate** | **SE** | **Lower** | **Upper** | **df** | **t** | **p** |
| --- | --- | --- | --- | --- | --- | --- | --- |
| (Intercept) | -0.000 | 0.126 | -0.247 | 0.247 | 55.000 | -0.002 | 0.998 |
| CCA_Cogn_mode2 | 0.354 | 0.126 | 0.108 | 0.601 | 55.000 | 2.816 | **0.007** |

CCAmode2 cognitive variate: Model 2

| **Names** | **Estimate** | **SE** | **Lower** | **Upper** | **df** | **t** | **p** |
| --- | --- | --- | --- | --- | --- | --- | --- |
| (Intercept) | -0.000 | 0.129 | -0.253 | 0.253 | 48.000 | -0.002 | 0.998 |
| Intracranial_Vol | -0.105 | 0.137 | -0.373 | 0.163 | 48.000 | -0.767 | 0.447 |
| Parental_educatoin | 0.134 | 0.156 | -0.171 | 0.439 | 48.000 | 0.860 | 0.394 |
| Sex | -0.213 | 0.251 | -0.705 | 0.279 | 48.000 | -0.849 | 0.400 |
| PHV | 0.356 | 0.327 | -0.285 | 0.997 | 48.000 | 1.089 | 0.282 |
| Age | -0.146 | 0.261 | -0.657 | 0.365 | 48.000 | -0.559 | 0.579 |
| SVI | 0.113 | 0.217 | -0.312 | 0.538 | 48.000 | 0.520 | 0.605 |
| School_Type | -0.103 | 0.205 | -0.504 | 0.299 | 48.000 | -0.501 | 0.619 |
| CCA_Cogn_mode2 | 0.363 | 0.132 | 0.105 | 0.622 | 48.000 | 2.752 | **0.008** |

Model 1: no adjusted; Model 2: Adjusted by age, PHV, sex, SVI, parent education, school type, and intracranial volume. Cluster: Schools (*k*=10). In bold significant values.

**Table S1F: FLICA 16 IC#9**

CCAmode2 physical variate: Model 1

| **Names** | **Estimate** | **SE** | **Lower** | **Upper** | **df** | **t** | **p** |
| --- | --- | --- | --- | --- | --- | --- | --- |
| (Intercept) | -0.022 | 0.153 | -0.322 | 0.278 | 4.646 | -0.142 | 0.893 |
| CCA_Beh_mode2 | 0.257 | 0.128 | 0.006 | 0.508 | 50.634 | 2.006 | **0.050** |

CCAmode2 physical variate: Model 2

| **Names** | **Estimate** | **SE** | **Lower** | **Upper** | **df** | **t** | **p** |
| --- | --- | --- | --- | --- | --- | --- | --- |
| (Intercept) | -0.042 | 0.174 | -0.383 | 0.300 | 2.345 | -0.240 | 0.829 |
| Intracranial_Vol | -0.103 | 0.136 | -0.369 | 0.162 | 45.864 | -0.762 | 0.450 |
| Parental_educatoin | 0.220 | 0.159 | -0.093 | 0.532 | 46.747 | 1.378 | 0.175 |
| Sex | -0.337 | 0.255 | -0.837 | 0.164 | 46.771 | -1.319 | 0.194 |
| PHV | 0.492 | 0.333 | -0.161 | 1.144 | 47.998 | 1.476 | 0.146 |
| Age | -0.223 | 0.262 | -0.737 | 0.290 | 47.916 | -0.852 | 0.398 |
| SVI | 0.180 | 0.291 | -0.390 | 0.749 | 2.295 | 0.618 | 0.592 |
| School_Type | -0.205 | 0.277 | -0.749 | 0.338 | 2.373 | -0.740 | 0.526 |
| CCA_Beh_mode2 | 0.418 | 0.143 | 0.138 | 0.697 | 47.366 | 2.930 | **0.005** |

Model 1: no adjusted; Model 2: Adjusted by age, PHV, sex, SVI, parent education, school type, and intracranial volume. Cluster: Schools (*k*=10). In bold significant values.

**Table S2A**. Mediation analysis for FLICA 14 IC#8.

|  | **Estimate** | **95% CI Lower to Upper** | **p** |
| --- | --- | --- | --- |
| Indirect effect | 0.126 | 0.022 to 0.280 | **0.010** |
| Direct Effect | 0.260 | -0.033 to 0.540 | 0.090 |
| Total effect | 0.386 | 0.086 to 0.680 | **0.010** |
| Prop. Mediated | 0.31 | 0.050 to 1.210 | **0.020** |

In bold significant values. Sample size: 57, Quasi-Bayesian Confidence Intervals, Simulations: 1000.

**Table S2B**. Mediation analysis for FLICA 16 IC#9.

|  | **Estimate** | **95% CI Lower to Upper** | **p** |
| --- | --- | --- | --- |
| Indirect effect | 0.075 | -0.004 to 0.200 | 0.076 |
| Direct Effect | 0.309 | 0.024 to 0.580 | **0.030** |
| Total effect | 0.385 | 0.094 to 0.680 | **0.008** |
| Prop. Mediated | 0.18 | -0.012 to 0.810 | 0.076 |

In bold significant values. Sample size: 57, Quasi-Bayesian Confidence Intervals, Simulations: 1000.
